# Meta‐analysis of the impact of postoperative infective complications on oncological outcomes in colorectal cancer surgery

**DOI:** 10.1002/bjs5.50302

**Published:** 2020-06-11

**Authors:** J. Lawler, M. Choynowski, K. Bailey, M. Bucholc, A. Johnston, M. Sugrue

**Affiliations:** ^1^ Department of Surgery Letterkenny University Hospital and Donegal Clinical Research Academy Donegal Ireland; ^2^ EU INTERREG Centre for Personalized Medicine, Intelligent Systems Research Centre, School of Computing, Engineering and Intelligent Systems Ulster University Magee Campus Derry /Londonderry UK

## Abstract

**Background:**

Cancer outcomes are complex, involving prevention, early detection and optimal multidisciplinary care. Postoperative infection and surgical site‐infection (SSI) are not only uncomfortable for patients and costly, but may also be associated with poor oncological outcomes. A meta‐analysis was undertaken to assess the oncological effects of SSI in patients with colorectal cancer.

**Methods:**

An ethically approved PROSPERO‐registered meta‐analysis was conducted following PRISMA guidelines. PubMed and Scopus databases were searched for studies published between 2007 and 2017 reporting the effects of postoperative infective complications on oncological survival in colorectal cancer. Results were separated into those for SSI and those concerning anastomotic leakage. Articles with a Methodological Index for Non‐Randomized Studies score of at least 18 were included. Hazard ratios (HRs) with 95 per cent confidence intervals were computed for risk factors using an observed to expected and variance fixed‐effect model.

**Results:**

Of 5027 articles were reviewed, 43 met the inclusion criteria, with a total of 154 981 patients. Infective complications had significant negative effects on overall survival (HR 1·37, 95 per cent c.i. 1·28 to 1·46) and cancer‐specific survival (HR 2·58, 2·15 to 3·10). Anastomotic leakage occurred in 7·4 per cent and had a significant negative impact on disease‐free survival (HR 1·14, 1·09 to 1·20), overall survival (HR 1·34, 1·28 to 1·39), cancer‐specific survival (HR 1·43, 1·31 to 1·55), local recurrence (HR 1·18, 1·06 to 1·32) and overall recurrence (HR 1·46, 1·27 to 1·68).

**Conclusion:**

This meta‐analysis identified a significant negative impact of postoperative infective complications on overall and cancer‐specific survival in patients undergoing colorectal surgery.

## Graphical abstract

Postoperative infective complications have significant negative effects on oncological outcomes in colorectal cancer. Anastomotic leakage has a negative effect on every measured outcome. New registries should include oncological outcomes and complications to facilitate future research and optimize outcomes.

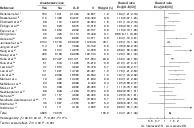

Colorectal cancer surgery infectious complications affect overall and cancer‐specific survival

## Abstract (es)

**Antecedentes:**

Los resultados del cáncer son complejos, implican prevención, detección precoz y atención multidisciplinaria óptima. La infección postoperatoria y la infección del sitio quirúrgico (*surgical site infection*, SSI) no solo son inconvenientes para los pacientes y costosas, sino que también pueden estar asociadas con malos resultados oncológicos. Este estudio realizó un metaanálisis para evaluar los efectos oncológicos de la SSI en pacientes con cáncer colorrectal.

**Métodos:**

Se realizó un metaanálisis registrado en PROSPERO, aprobado por el comité ético, siguiendo las pautas de PRISMA y utilizando las bases de datos PubMed y Scopus para estudios entre 2007‐2017 que describían los efectos de las complicaciones infecciosas postoperatorias en la supervivencia oncológica en el cáncer colorrectal. Los resultados se separaron para el grupo de infección del sitio quirúrgico (SSI) y de fuga anastomótica. Se incluyeron los artículos con una puntuación ≥ 18 según el índice MINORS. Para los factores de riesgo se calcularon los cocientes de riesgos instantáneos (*hazard ratios*, HR) mediante un modelo de efectos aleatorios y el método de Mantel‐Haenszel con los i.c. del 95% utilizando el programe RevMan5.

**Resultados:**

Se revisaron 5.027 artículos de los cuales 43 cumplieron con los criterios de inclusión. En total fueron 154.981 pacientes en los cuales las complicaciones infecciosas tuvieron efectos negativos significativos en la supervivencia global (HR: 1,37 i.c. del 95%: 1,28‐1,46) y la supervivencia específica relacionada con el cáncer (HR: 2,58 i.c. del 95%: 2,15‐3,10). La fuga anastomótica ocurrió en un 7,4% de los casos e impactó negativa y significativamente en la supervivencia libre de enfermedad (HR: 1,14 i.c. del 95%: 1,09‐1,20), en la supervivencia global (HR: 1,34 i.c. del 95%: 1,28‐1,39), en la supervivencia específica relacionada con el cáncer (HR: 1,43 i.c. del 95% 1.31‐1.55), en la recidiva local (HR: 1,18 i.c. del 95%: 1,06‐1,32) y en la recidiva global (HR: 1,46 i.c. del 95%: 1,27‐1,68).

**Conclusión:**

Este metaanálisis identificó un impacto negativo significativo en la supervivencia global y en la supervivencia específica relacionada con el cáncer en pacientes con complicaciones postoperatorias infecciosas sometidos a cirugía colorrectal.

## Introduction

Colorectal cancer affects 17 people per 100 000 worldwide and 30 per 100 000 in Europe^1^, with an average 5‐year survival rate of 65 per cent^2^. Optimizing cancer outcomes is a complex interaction involving key strategies: prevention, early detection and optimal management^3^. Many treatment paradigm shifts in both surgical and oncological treatment have improved cancer outcomes. Recurrence, which affects over 40 per cent of patients, has classically been associated with tumour stage, grade, emergency presentation and resection margin status^4^, ^5^.

Surgical‐site infections (SSIs), including superficial, deep and organ space infections, are coming increasingly under the spotlight, causing discomfort for patients and family, anxiety for surgeons, and cost to healthcare systems^6^. In addition, they are associated with potential delay in, or omission of, adjuvant therapy.

A recent long‐term analysis from the German Rectal Cancer Trial^7^ suggested that surgical complications were associated with both oncological and overall outcomes. Immunological forces influence survival^8^. As SSI occurs in approximately 15 per cent of patients undergoing colorectal surgery, a clear understanding of any adverse relationship is important^9^.

Although surgeons and patients alike fear the morbidity and mortality associated with postoperative complications, their potential negative impact on oncological outcomes is not widely understood or reported routinely^10^, ^11^. A meta‐analysis was undertaken to determine the impact of postoperative infections on oncological outcomes in colorectal cancer surgery.

## Methods

A study was conducted to assess the impact of postoperative infective complications on oncological outcomes in colorectal cancer surgery. The study was registered with PROSPERO (registration number: 42017069038) and followed PRISMA guidelines^12^. PubMed and Scopus were searched for studies that met the eligibility criteria. Original articles, published between June 2007 and May 2017, which reported the effect of infective complications on oncological survival in both colonic and rectal cancer were identified. The search strategy used the following keywords: Colon Cancer, Colorectal Cancer, Rectal Cancer, Complication, Infection, Oncological Outcomes, Anastomotic Leak, Survival and SSI. Animal studies, review articles, non‐English papers, duplicate data sets and results published only in abstracts were excluded. Details of the search strategy and data management are available in *Tables* *S1* and *S2* (supporting information).

### Data extraction and quality assessment

The abstracts were screened by one author and full texts by three authors. The descriptive and quantitative data from the screened studies were extracted and papers were graded using the Methodological Index for Non‐Randomized Studies (MINORS)^13^. The MINORS criteria have been designed to assess the quality of comparative and non‐comparative surgical studies using a three‐point scale (0, not reported; 1, reported but inadequate; 2, reported and adequate), with assessment of eight items for non‐comparative studies and 12 items for comparative studies. The ideal global scores for comparative and non‐comparative studies are 24 and 16 respectively.

Articles were graded by three reviewers initially, and only those that scored at least 18 of 24 were included in the statistical analysis. If there was disagreement on whether a paper should be included or not, another reviewer graded it and made the final decision. At the outset both rectal and colonic cancer procedures were grouped into a single category.

Results were separated into two key categories: infective complications (SSI, organ space infections, infectious complications, sepsis) and anastomotic leakage. SSI was defined according to the Centers for Disease Control and Prevention^14^ definition, whereas anastomotic leak was defined as reported in each article.

Overall survival, disease‐free survival, cancer‐specific survival and cancer recurrence data were analysed for each outcome where data were available and applicable. Survival terms were defined in accordance with National Institutes of Health–National Cancer Institute definitions^15^.

### Statistical analysis

For oncological outcomes, hazard ratios (HRs) were taken from papers or calculated using the MedCalc® statistical calculator (MedCalc, Ostend, Belgium). Observed minus expected (O‐E) values and variance were calculated^16^, and used to compute statistical values for use in the analysis.

Statistical analysis was performed in Review Manager (RevMan) version 5 (Nordic Cochrane Centre, Cochrane Collaboration, Copenhagen, Denmark) using O‐E and variance, a fixed‐effect model for analysis and HR as effect measure, with 95 per cent confidence intervals. Significance was assessed at the two‐sided 5 per cent level using HRs. The complication has a significant effect on the measured oncological outcome if the 95 per cent confidence interval of the HR does not include 1·00.

## Results

**Figure 1:**
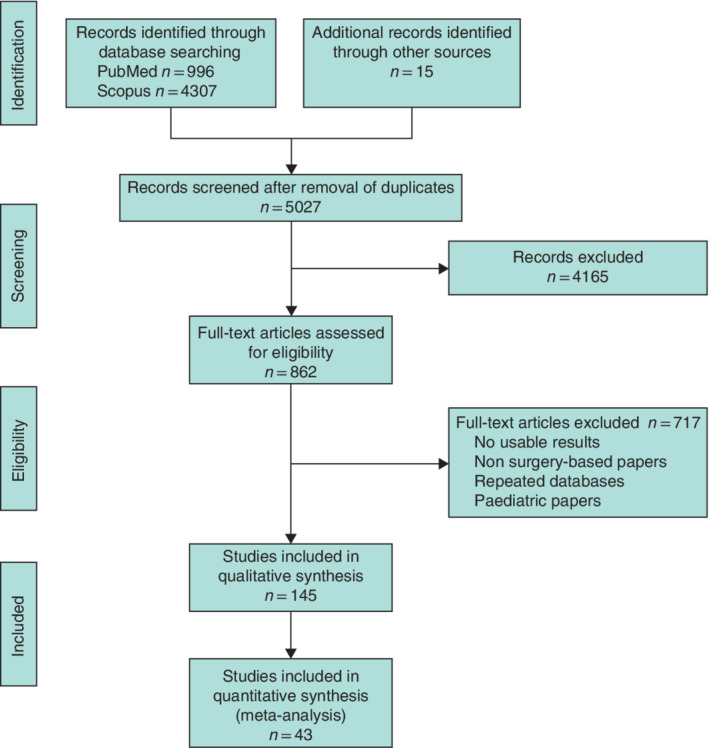
PRISMA flow chart showing selection of articles for review

A total of 5027 individual articles were reviewed in this study (*Fig*. *1*), of which 145 were found to be relevant and underwent MINORS grading. Forty‐three articles^17^, ^18^, ^19^, ^20^, ^21^, ^22^, ^23^, ^24^, ^25^, ^26^, ^27^, ^28^, ^29^, ^30^, ^31^, ^32^, ^33^, ^34^, ^35^, ^36^, ^37^, ^38^, ^39^, ^40^, ^41^, ^42^, ^43^, ^44^, ^45^, ^46^, ^47^, ^48^, ^49^, ^50^, ^51^, ^52^, ^53^, ^54^, ^55^, ^56^, ^57^, ^58^, ^59^ met all inclusion criteria and were used in the data analysis, with a total cohort size of 154 981 patients (*Table* *1*). Publications were from the USA (7), Korea (5), the UK (4), Japan (4), China (4), Germany (4) and other countries (15). There were 23 retrospective and 20 prospective studies in this meta‐analysis. Ten studies were from multicentre databases (6 prospective, 4 retrospective).

**Table 1:** Study characteristics

Reference	Country	Study design	Multicentre database study	No. of patients	Anastomotic leak
Bertelsen *et al*.^17^	Denmark	Prospective	Yes	1494	163 (10·9)
Cone *et al*.^18^	USA	Prospective	Yes	24 730	
Espín *et al*.^19^	Spain	Prospective	Yes	1181	100 (8·5)
Jörgren *et al*.^20^	Sweden	Prospective	Yes	1977	172 (8·7)
Krarup *et al*.^21^	Denmark	Prospective	Yes	9333	593 (6·4)
Kube *et al*.^22^	Germany	Prospective	Yes	28 271	844 (3·0)
Aquina *et al*.^23^	USA	Retrospective	Yes	24 426	
Artinyan *et al*.^24^	USA	Retrospective	Yes	12 075	
Chu *et al*.^25^	USA	Retrospective	Yes	528	
Nordholm‐Carstensen *et al*.^26^	Denmark	Retrospective	Yes	774	71 (9·2)
Boccola *et al*.^27^	Australia	Prospective	No	1576	110 (7·0)
Duron *et al*.^28^	France	Prospective	No	3322	
Eberhardt *et al*.^29^	USA	Prospective	No	177	59 (33·3)
Gong *et al*.^30^	China	Prospective	No	460	35 (7·6)
Gupta *et al*.^31^	Nepal	Prospective	No	272	18 (6·6)
Jannasch *et al*.^32^	Germany	Prospective	No	17 867	2134 (11·9)
Law *et al*.^33^	China	Prospective	No	1657	47 (2·8)
Law *et al*.^34^	China	Prospective	No	1580	60 (3·8)
Platt *et al*.^35^	UK	Prospective	No	454	
Ptok *et al*.^36^	Germany	Prospective	No	2044	303 (14·8)
Richards *et al*.^37^	UK	Prospective	No	423	18 (4·3)
Smith *et al*.^38^	USA	Prospective	No	1127	40 (3·5)
Smith *et al*.^39^	USA	Prospective	No	184	12 (6·5)
Thorgersen *et al*.^40^	Norway	Prospective	No	540	
Attiê *et al*.^41^	Brazil	Retrospective	No	106	
Ebinger *et al*.^42^	Switzerland	Retrospective	No	584	64 (11·0)
Goto *et al*.^43^	Japan	Retrospective	No	3364	85 (2·5)
Haruki *et al*.^44^	Japan	Retrospective	No	77	
Huang *et al*.^45^	China	Retrospective	No	215	
Jung *et al*.^46^	Korea	Retrospective	No	1391	35 (2·5)
Kang *et al*.^47^	Korea	Retrospective	No	1083	69 (6·4)
Katoh *et al*.^48^	Japan	Retrospective	No	1101	
Kerin Povšič *et al*.^49^	Slovenia	Retrospective	No	186	
Kulu *et al*.^50^	Germany	Retrospective	No	570	51 (8·9)
Lee *et al*.^51^	Korea	Retrospective	No	1278	51 (4·0)
Lim *et al*.^52^	Korea	Retrospective	No	2510	141 (5·6)
Marra *et al*.^53^	Switzerland	Retrospective	No	445	12 (2·7)
McMillan *et al*.^54^	UK	Retrospective	No	920	24 (2·6)
Miccini *et al*.^55^	Italy	Retrospective	No	479	34 (7·1)
Mrak *et al*.^56^	Austria	Retrospective	No	811	54 (6·7)
Nachiappan *et al*.^57^	UK	Retrospective	No	1048	99 (9·4)
Noh *et al*.^58^	Korea	Retrospective	No	1258	101 (8·0)
Tsujimoto *et al*.^59^	Japan	Retrospective	No	1083	29 (2·7)
					
Total				154 981	7·4 (2·5–33·3)%*

Values in parentheses are percentages unless indicated otherwise; *values are mean (range).

### Non‐anastomotic infective complications

**Figure 2:**
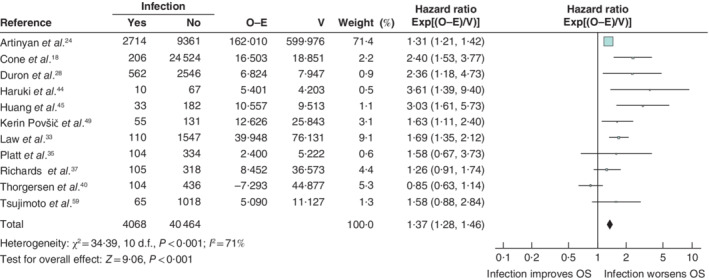
Impact of surgical‐site infection on overall survival Hazard ratios are shown with 95 per cent confidence intervals. A fixed‐effect model was used for meta‐analysis. O‐E, observed to expected; V, variance; OS, overall survival.

Sixteen papers reported SSI data that allowed meaningful analysis. Of these, 11 of 16 papers contained data on overall survival. Three^37^, ^40^, ^44^ of 11 articles reported disease‐free survival and two^23^, ^41^ of 11 articles cancer‐specific survival. Infective complications were shown to have a significant negative effect on overall survival (HR 1·37, 95 per cent c.i. 1·28 to 1·46) (*Fig*. *2*) and cancer‐specific survival (HR 2·58, 2·15 to 3·10). However, there was no significant association between infective complications and disease‐free survival (HR 0·89, 0·74 to 1·08).

### Anastomotic leakage

Anastomotic leakage data were suitable for analysis in 31 publications. The mean leak rate was 7·4 (range 2·5–33·3) per cent (*Table* *1*). The effect of anastomotic leakage on overall survival could be assessed in 24 articles, and its effect on disease‐free survival in ten of 31 studies. Cancer‐specific survival was reported in ten of 31 articles. Nineteen of the 31 articles reported on local recurrence and ten on overall recurrence.

**Figure 3:**
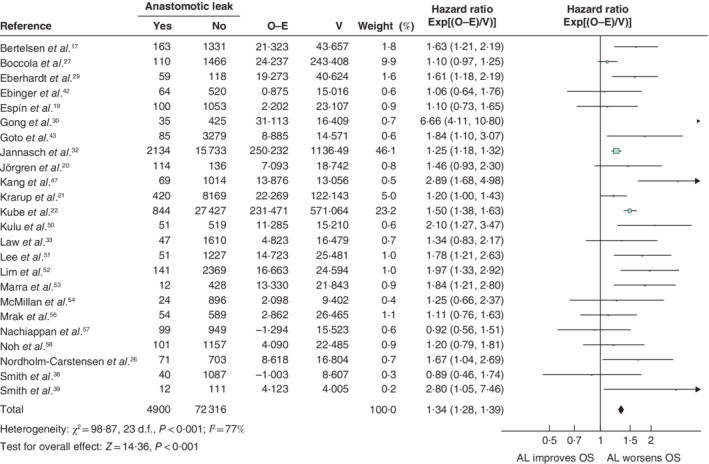
Impact of anastomotic leakage on overall survival Hazard ratios are shown with 95 per cent confidence intervals. A fixed‐effect model was used for meta‐analysis. O‐E, observed to expected; V, variance; OS, overall survival.

**Figure 4:**
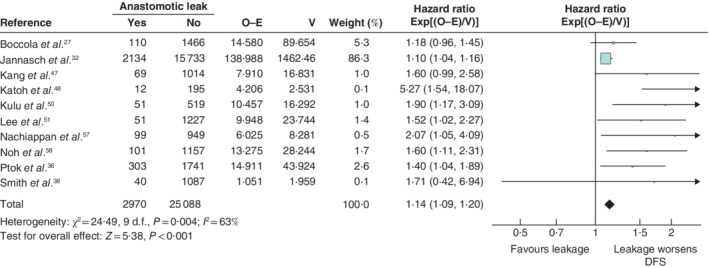
Impact of anastomotic leakage on disease‐free survival Hazard ratios are shown with 95 per cent confidence intervals. A fixed‐effect model was used for meta‐analysis. O‐E, observed to expected; V, variance; AL, anastomotic leak; DFS, disease‐free survival.

**Figure 5:**
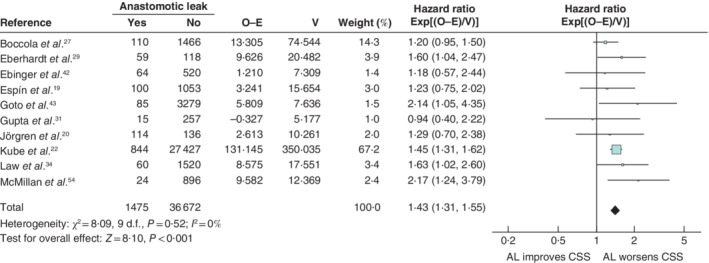
Impact of anastomotic leakage on cancer‐specific survival Hazard ratios are shown with 95 per cent confidence intervals. A fixed‐effect model was used for meta‐analysis. O‐E, observed to expected; V, variance; AL, anastomotic leak; CSS, cancer‐specific survival.

**Figure 6:**
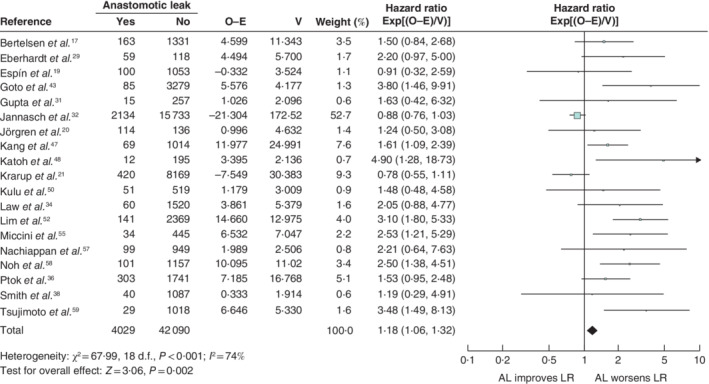
Impact of anastomotic leakage on local recurrence Hazard ratios are shown with 95 per cent confidence intervals. A fixed‐effect model was used for meta‐analysis. O‐E, observed to expected; V, variance; AL, anastomotic leak; LR, local recurrence.

**Figure 7:**
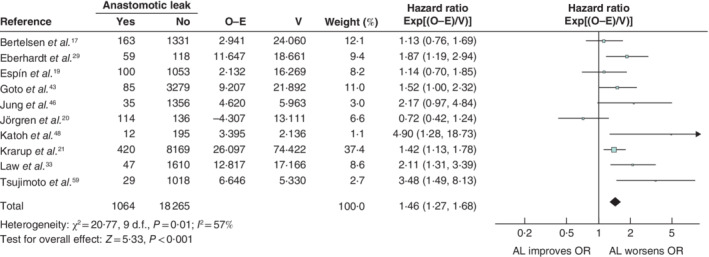
Impact of anastomotic leakage on overall recurrence Hazard ratios are shown with 95 per cent confidence intervals. A fixed‐effect model was used for meta‐analysis. O‐E, observed to expected; V, variance; AL, anastomotic leak.

Anastomotic leakage had a negative impact on overall survival (HR 1·34, 95 per cent c.i. 1·28 to 1·39) (*Fig*. *3*), disease‐free survival (HR 1·14, 1·09 to 1·20) (*Fig*. *4*), cancer‐specific survival (HR 1·43, 1·31 to 1·55) (*Fig*. 5), local recurrence (HR 1·18, 1·06 to 1·32) (*Fig*. *6*) and overall recurrence (HR 1·46, 1·27 to 1·68) (*Fig*. *7*).

## Discussion

This meta‐analysis of 154 981 patients in 43 studies evaluated the impact of both wound‐related non‐anastomotic infective complications and anastomotic leakage, and identified a statistically significant negative oncological effect.

From the outset of this extensive literature review there were a number of limitations. In the overall cohort, narrowed by the quality of data and MINORS analysis, there was significant heterogeneity. SSI definitions are problematic, with variation from study to study. This is unfortunately common in all forms of surgery. In a 20‐year period up to 2015, only 18 per cent of the top 50 cited peer‐reviewed publications on ventral hernia were found to use a standardized definition of SSI and surgical‐site occurrence after ventral hernia repair^60^, ^61^. The absence of a common language impedes comparisons in the literature and accurate metrics of hospital quality measures^60^. In addition, the period of surveillance used to report SSI varies between 30 and 60 days^42^, ^60^. Anastomotic leak itself has a heterogeneous spectrum of presentation, depending on the effort made to detect leakage and the criteria used, whether based on combined clinical, radiological or endoscopic features. This may give rise to heterogeneity representing a potential limitation of this meta‐analysis. Few articles, in general, addressed the effect of SSI on oncological outcomes; some evaluated overall survival, a few reported disease‐free survival and none considered the recurrence rate. Furthermore, owing to the limited numbers of papers, it was not possible to undertake a subset analysis for different stages of colorectal cancer, nor to differentiate between colonic and rectal cancers.

The mean leak rate was 7·4 per cent across the 31 articles included in the analysis of anastomotic leak; this is in keeping with the mean leak rate in international data^62^. Anastomotic leakage is increasingly topical; there have been paradigm shifts in surgical, prehabilitation, intraoperative and postoperative approaches to reducing leakage^62^, ^63^, ^64^.

This meta‐analysis reinforces the findings of a meta‐analysis^65^ in 2016, which showed that complication severity had a significant impact on both disease‐free and overall survival. Three other studies^66^, ^67^, ^68^ identified a negative impact of anastomotic leakage on long‐term cancer‐specific survival, particularly noting an increase in local recurrence. Current efforts at SSI management after colorectal surgery focus on compliance with guidelines and evaluation of infection rates, but Gantz and colleagues^69^ recently suggested that improvement is needed. Martinez *et al*.^70^ suggested establishing national SSI bundles. Historically, mechanical and oral bowel preparations were favoured, but then bowel preparation went out of vogue. Now there is the potential for reintroduction of bowel cleansing and recognition of the importance of other factors including those relating to the gut microbiome. The gut microbiome potentially has an effect on infection and also a separate oncological effect. A variety of environmental factors, including diet, antibiotics, bowel preparation and surgical stress, act on the microbiome, altering its architecture and function, with a negative effect on oncological outcomes after surgery^71^. It is clear from the present data that anastomotic leakage is associated with increased local recurrence and decreased overall survival. The recent German rectal trial CAO/ARO/AIO‐94^7^ showed that surgical complications are significantly associated with reduced overall survival. Patients with complications are more likely to have distant metastasis and local recurrences. The reason for this is somewhat unclear, although it is known that cancer cells shed from the bowel may embed themselves on stapling devices, leading to enhanced tumour dissemination in the event of anastomotic leak or reoperation. Exfoliated cancer cells have been detected in the colonic lumen and on stapling devices, suggesting that anastomotic leakage could enhance dissemination^72^, ^73^.

There are many confounders to the potential negative oncological effects of infection. Systemic inflammation has been shown to promote micrometastasis^74^. An infection‐led inflammatory cascade will activate cytokines, and cell‐ and humoral‐mediated immunity.

Local recurrence is an important clinical outcome for patients with colorectal cancer; many treatment modalities have been investigated with the aim of reducing pelvic occurrence from total mesorectal excision to neoadjuvant chemoradiotherapy. The present study has identified that additional measures and routine use of SSI prevention bundles need to be implemented to reduce infective complications^75^. Infection prevention should become a potential target for oncological improvement; opportunities to reduce deep wound infection need to be revisited, incorporating wound bundles, intraoperative protective measures such as use of wound protectors, potential antibiotic solution and rectal washouts, and closer monitoring with intra‐abdominal pressure measurement after surgery.

This study had a number of limitations. An initial trawl of the literature identified almost 13 000 potential publications. On deeper analysis, including qualitative evaluation using the MINORS criteria, it was found that many of these papers lacked a definition of either SSI or anastomotic leakage^60^, ^61^ and, most importantly, no relationship between adverse events and oncological outcome was reported. In contrast, it is increasingly being recognized in other fields of oncology, such as breast cancer, that there may be a relationship between infection and cancer recurrence^76^. Surprisingly SSI data have not been included in cancer registries. Uniform data definitions and data analysis would make analysis easier. The small number of papers reporting infective complications may have led to bias in the present results. Subset analysis of SSI effects at different cancer stages was not possible.

This meta‐analysis has identified a statistically significant association between both anastomotic leak and wound infection/SSI and adverse oncological outcomes. Oncological registries incorporating infective and adverse events as part of their outcome analysis may help in understanding the relationship between SSI and oncological outcomes. Reduction in SSI may prove to be a noteworthy part of adjuvant cancer therapy, and wound bundles should become mandatory. There needs to be greater adoption and monitoring of strategies that might reduce SSIs and their negative impact.

## Acknowledgements

M.B. is supported by the European Union's INTERREG VA Programme, managed by the Special EU Programmes Body (SEUPB) and the Dr George Moore Endowment for Data Science at Ulster University.

*Disclosure:* The authors declare no conflict of interest.

## Supporting information


**Table S1** **Advanced search strategies and Boolean characters used across the various databases**
Table S2 **Data Management**
