# Supplementary material for: Meta‐analysis of the impact of postoperative infective complications on oncological outcomes in colorectal cancer surgery
Source: BJS Open. 2020 Jun 11;4(5):737–47. doi: 10.1002/bjs5.50302 (PMC7528523; doi:10.1002/bjs5.50302)
Supplement: Supplementary file 1 — Table S1 Advanced search strategies and Boolean characters used across the various databases Table S2 Data Management [file BJS5-4-737-s001.docx]

**BJS5_50302**

**Meta-analysis of the impact of postoperative infective complications on oncological outcomes in colorectal cancer surgery**

J. Lawler, M. Choynowski, K. Bailey, M. Bucholc, A. Johnston and M. Sugrue

**Table S1 Advanced search strategies and Boolean characters used across the various databases**

| Search Type | Limits | Search Terms Used  (MESH Term Searching enabled) | Number of Results | |
| --- | --- | --- | --- | --- |
|  |  |  | PubMed | Scopus |
| Advanced | PubMed: 2007/06/01 to 2017/05/31  Scopus: 2007 to 2017 | ((Colon Cancer) AND Complication) AND Oncological Outcome | 38 | 299 |
| Advanced | PubMed: 2007/06/01 to 2017/05/31  Scopus: 2007 to 2017 | ((Colon Cancer) AND Infection) AND Oncological Outcome | 10 | 218 |
| Advanced | PubMed: 2007/06/01 to 2017/05/31  Scopus: 2007 to 2017 | ((Colorectal Cancer) AND Complication) AND Oncological Outcome | 91 | 354 |
| Advanced | PubMed: 2007/06/01 to 2017/05/31  Scopus: 2007 to 2017 | ((Colorectal Cancer) AND Infection) AND outcome | 423 | 1,604 |
| Advanced | PubMed: 2007/06/01 to 2017/05/31  Scopus: 2007 to 2017 | ((Colorectal Cancer) AND SSI) AND outcome | 41 | 170 |
| Advanced | PubMed: 2007/06/01 to 2017/05/31  Scopus: 2007 to 2017 | (Anastomotic leak) AND oncological outcome | 67 | 211 |
| Advanced | PubMed: 2007/06/01 to 2017/05/31  Scopus: 2007 to 2017 | (Anastomotic leak) AND survival AND rectal cancer | 247 | 443 |
| Advanced | PubMed: 2007/06/01 to 2017/05/31  Scopus: 2007 to 2017 | (Colorectal Cancer) OR (Rectal Cancer) AND Infection OR SSI AND Oncological Outcome | 43 | 435 |
| Advanced | PubMed: 2007/06/01 to 2017/05/31  Scopus: 2007 to 2017 | (Rectal Cancer) AND SSI AND outcome | 19 | 76 |
| Advanced | PubMed: 2007/06/01 to 2017/05/31  Scopus: 2007 to 2017 | (Rectal Cancer) AND SSI AND survival | 8 | 19 |
| Advanced | Clinical Trials Only  PubMed: 2007/06/01 to 2017/05/31  Scopus: 2007 to 2017 | Colorectal cancer AND complication AND Survival | 53 | 2,082 |
| Advanced | PubMed: 2007/06/01 to 2017/05/31  Scopus: 2007 to 2017 | Colorectal cancer AND SSI AND survival | 18 | 58 |

Databases accessed June 2017. Out of range Scopus searches removed in Excel post compilation of papers

**Table S2 Data Management**

| PubMed: Send to > File > CSV > Import to Excel | Scopus: Select All > Export > CSV Export > Import to Excel |
| --- | --- |
| PubMed results collated in single Excel file | Scopus results collated in single Excel file |
| Duplicates removed via Data>Removed Duplicates | Duplicates removed via Data>Removed Duplicates |
| Remove inapplicable result types via sorting by Type   - Letter/Note/Meta-analysis/Survey |  |
| Scopus/PubMed post-duplication Excel files combined into single Excel spreadsheet | |
| Arrange columns to match due to CSV format differences | |
| Duplicates removed via Data>Removed Duplicates   - Keeping PubMed papers | |
| Foreign Language papers removed by removing [] bracketed titles | |
| Key word in title removal of papers  Meta-analysis  Systematic Review  A case report  Letter to Editor  Paediatric  Palliative  Hernia  Phase 1 trial | |
| Remaining papers were assessed via title/abstract reading for relevance and usable results | |
